# Endoparasites of Jungle Cats (*Felis chaus*) and Their Pathologic Lesions

**Published:** 2018

**Authors:** Seyed Reza TABARIPOUR, Mohammad Reza YOUSSEFI, Seyed Mohammad HOSSEINI

**Affiliations:** 1. Dept. of Cellular and Molecular, Babol Branch, Islamic Azad University, Babol, Iran; 2. Dept. of Veterinary Parasitology, Babol Branch, Islamic Azad University, Babol, Iran; 3. Dept. of Pathology, Babol Branch, Islamic Azad University, Babol, Iran

**Keywords:** *Toxocara cati*, *Alaria alata*, *Mesocestoides lineatus*, *Felis chaus*, Iran

## Abstract

**Background:**

The jungle cats (*Felis chaus*) are native to Asia, and found in Iran. Although north of Iran has a wide distribution of jungle cats, there is not any data about prevalence of parasitic infections in the population of these cats.

**Methods:**

From 2012 to 2015, seven specimens of the wild jungle cat (*Felis chaus*) from north of Iran, Mazandaran Province, northern Iran were collected and examined for their endoparasites and pathological lesions which were caused by the parasites.

**Results:**

Parasitological evaluations showed several species of endoparasites in the small intestine of cats including nematode (*Toxocara cati*), trematode (*Alaria alata*), and cestode (*Mesocestoides lineatus*). All of the examined jungle cats were infected to parasitic infections. From the total number of 7 jungle cats *T. cati* was found in 6 cats, *A. alata* in 1 cat, and *M. lineatus* was recovered from 3 cats. Histopathological samples displayed necrosis, vacuolar degeneration, atrophy, destruction of tissue, hyperemia, and infiltration of inflammatory cells in the intestine tissue.

**Conclusion:**

This study for the first reported *T. cati*, *A. alata,* and *M. lineatus* and their pathologic effects in the small intestine of jungle cat.

## Introduction

The jungle cats (*Felis chaus*) are native to Asia from southern China in the east through southeast, west, central Asia to the Nile valley in the west and also in South Asia (1). It is listed as least concern in the International Union for Conservation of Nature (IUCN) red list of threatened species as it is widespread and common, particularly in India (2) and according to the anecdotal information they have numerous populations in the north of Iran. Female jungle cats are slightly smaller than males, but average range is 50 to 94 cm in length, and weight varies across their range from 3 to 16 kg, with a median weight of around 8 kg (3). They have different colors in different species including tawny-grey, yellowish-grey to reddish-brown ticked with black (4). Jungle cats are single in nature and rest in other animals’ shelters, or under rocks, or in ground holes in areas with dense vegetation. They are active at night; their locomotion have been considered from 3 to 6 km per night depending on the accessibility of prey (2). They are carnivorous mainly on birds, squirrels, rodents, hares, frogs, as well as various reptiles including snakes and turtles; many diseases such as parasitic infections could be transferred to jungle cats through their feed animals (5).

*A. alata* has been founded in wildlife and members of canidae, felidae, and mustelidae families. Miracidia and cercaria stages of this parasite use aquatic snails and amphibians, respectively as intermediate hosts, and adult parasites are located in the small intestines of carnivores. Although many studies showed pathologic effects of *A. alata* infection in humans, studies on effect of this parasite on animals are very limited (6).

*T. cati* is one of the common dangerous nematodes of felidae and has a cosmopolitan distribution (7). Toxocariasis is the disease caused by this parasite which is usually asymptomatic (8). Adult worms of *T. cati* are localized in the small intestine and produce eggs. Felines can be infected by three routes, ingestion of rodents containing larvae, ingestion of parasite eggs that found in contaminated soil or transmammary infection of kittens (9). Adult worms cause pathologic effects in the small intestine tissue resulting in catarrhal enteritis and diarrhea (9, 10).

*M. lineatus* is a rare parasitic intestinal tape-worm which infects cats (11). The life cycle of this parasite has not been completely known, but probably mite or a coprophillic insect ingests the egg and cysticercoid matures in the arthropods. Cysticercoid develops to a tetrathyridium when the second host (rodents, opossums, amphibians, reptiles, birds, and mammals) ingests infected arthropods and if cats accidentally ingest the second intermediate host, tetrathyridia will develop into adult cestodes (12, 13). Adult of this parasite occurs in the small intestine; when a cat is infected with this parasite, enteritis, peritonitis, and ascites may occur (14).

Although northern provinces of Iran have a wide distribution of jungle cats, there is not enough published data on the prevalence of helminthic infection and their pathologic effects in the population of these cats. So the current study aimed to study the endoparasitic infections and their pathologic effects in the jungle cats of the north of Iran.

## Materials and Methods

### Sampling

During a three-year period (December 2012 to May 2015), a total number of seven jungle cats (3 females and 4 males) killed by road accidents or recorded from illegal hunters by Mazandaran Department of Environment were transferred to the Faculty of Veterinary Medicine of Islamic Azad University of Babol Branch, Babol, Iran. Ethics Committee of the university approved the study.

For necropsy examinations and searching for parasites and pathological lesions, all internal organs (oral cavity, oesophagus, stomach, intestines, trachea, lung, liver, kidneys, gall-bladder, urinary bladder, and bile ducts) were checked. Several parsites were recovered from intestine.

Visible helminths of intestine were screened separately by Mesh 70 and samples were transferred to Petri dishes. Trematode and cestode samples were fixed and preserved in 70% ethanol, stained with carminic acid, dehydrated then cleared and mounted in Canada-balsam (Merk, Germany). Nematodes specimens were killed in hot saline solution, fixed in a solution made with 70% ethanol and 5% glycerin then cleared by a droplet of lactophenol and mounted by Canada-balsam (Merk, Germany). Recognition of helminthes was performed by a stereo-microscope (Olympus, Japan) and according to available systematic keys (15–17). For histopathological examinations, serial sections of tissue samples were taken from the attachment place of the parasites to the intestine. All of the samples were transferred to 10% buffered formalin and serial sections of formalin-fixed, paraffin-embedded of samples were produced and stained by routine Hematoxylin and Eosin (H&E).

### Study area

Mazandaran (latitude 36.5656° N, longitude 53.0588° E) is a province in the north of Iran. This province has a particular geographical condition with moderate and subtropical climate, coastal plains, mountainous areas and diverse ecosystems prairies and forests.

## Results

**Fig. 1:**
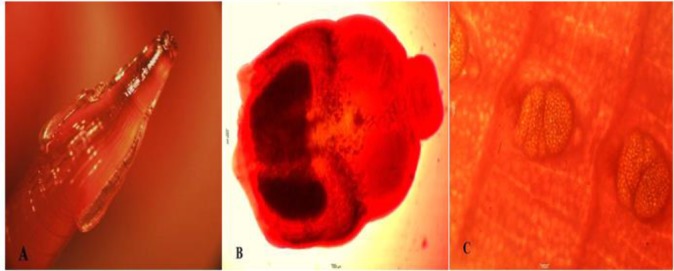
Helminths recovered from jungle cats of Mazandaran province, Iran. (A) *Toxocara cati*: Head of *T. cati* has cervical alae and the distinct appearance of an arrow. (B) *Alaria alata*: (C) *Mesocestoides lineatus*.

Seven jungle cats (3 females, 4 males) were examined for their internal parasites. Several parasites were recovered including nematodes, trematodes, and cestodes recognized as *T. cati*, *A. alata,* and *M. lineatus*, respectively (Fig. 1).

Six out of seven jungle cats (2 females and 4 males), one out of seven (1 female) and three out of seven jungle cats (2 females and 1 males) were infected with *T. cati*, *A. alata* and *M lineatus*, respectively.

**Fig. 2:**
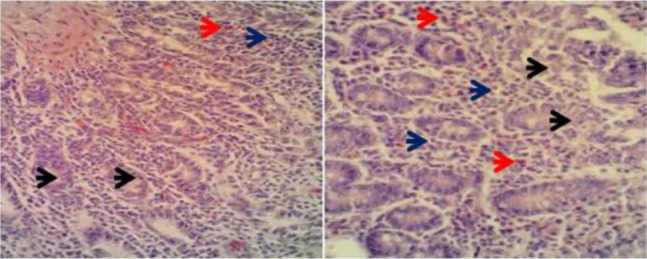
Infected small intestine tissue of jungle cat to *Toxocara cati*. Necrosis (*black arrows*) inflammatory cells infiltration [eosinophils (*red arrows*), lymphocytes (*blue arrows*)]. H&E staining. ×40 magnification.

**Fig. 3:**
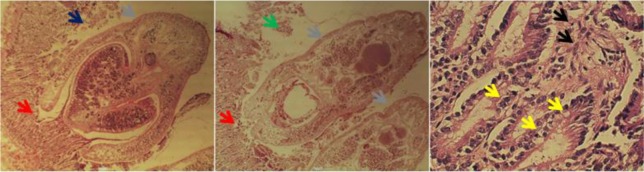
Infected small intestine tissue of jungle cat to *Alaria alata*. Section of parasite (*gray arrows*), necrosis (*black arrows*), tissue distraction (*blue arrows*), atrophy (*red arrows*), inflammatory cells infiltration (*green arrows*), vacuolar degeneration (*yellow arrows*). H&E staining. ×10, 40 magnification

**Fig. 4:**
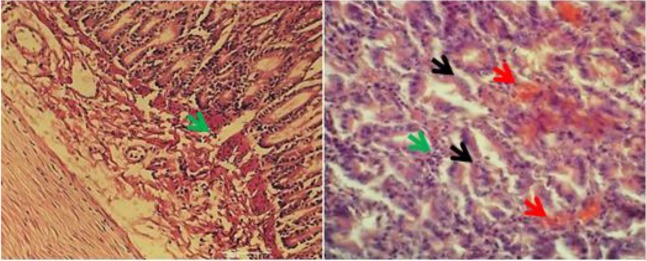
Infected small intestine tissue of jungle cat to *Mesocestoides lineatus*. Necrosis (black arrows), hyperemia (red arrows), inflammatory cells infiltration (green arrows). H&E staining. ×10, 40 magnification.

Microscopic examination of *T. cati* histo-pathological samples showed necrosis, infiltration of inflammatory cells including eosinophils and lymphocytes (Fig. 2). Tissue necrosis, inflammatory cells infiltration, mucosal atrophy, and vacuolar degeneration were observed in histopathological samples of *A. alata* (Fig. 3). Necrosis, hyperemia, and infiltration of inflammatory cells (including lymphocytes) were pathological effects caused by *M. lineatus* in small intestine tissue (Fig. 4).

## Discussion

Jungle cats have a wide distribution in Asia and Iran. They feed intermediate hosts of different parasites and as a result different types of parasitic infections can be seen in this species. Habitat preferences and diet are among factors that affect the prevalence of helminthic parasites in jungle cats. The higher parasite burden in wild cats in comparison to the domestic cats can be described by the broader spectrum of prey species as potential intermediate hosts in their diet (18).

The present study, for the first time has reported the parasitic infections of jungle cats of northern Iran. *T. cati, A. alata,* and *M. lineatus*. There has not been any previous report about these parasites and their pathologic effects in the jungle cats of Iran, but *T. cati* was previously reported in wild felidae of Thailand (19), from stray cats in northern Iran (20), and from wild cats in Germany (21). Moreover, *A. alata* was reported from wild canis and felis in Argentina and Brazil (22, 23), in red foxes in Ireland (24, 25), and in French wild boars populations. *M. lineatus* was found in jungle cats in Iraq (26) and wide range of wild carnivores (dogs and cats) (27).

Higher prevalence of helminths was reported in males and the reasons for the male-biased parasitism were behavioral, territorial, hormone, immune system, movement and diets (28). However, there was no significant difference between number of parasites recovered from male and female jungle cats. A study showed 87% rate of parasitic infections in wild cats from Slovakia and another one reported an endoparasitic infection rate of 93% in wild cats (n=15) observed. In the present study, all of the jungle cats were affected to parasitic infection and any cat without any parasite was not observed. In this study, parasitic infection rate of *T. cati*, *A. alata* and *M. lineatus* were 6/7, 1/7, 3/7, respectively. The most prevalent parasite was nematode (*T. cati*) and the least prevalent was trematode (*A. alata*). In a similar study on jungle cats, most prevalent parasites were cestodes (*Taenia crassiceps* and *Mesocestoides* sp.) and least prevalent parasite was Acanthocephala (*Oncicola* prob*. travassosi*). Most nematode species in wild cats were *Toxocara mystax* (73%) fallowed by *Toxacaris leonina* (60%) and most found cestode species was *Taenia taeniaeformis* (53%) (21, 29).

In the current survey, histopathological changes in the small intestine of infected jungle cats were necrosis, vacuolar degeneration, atrophy, hyperemia and inflammatory cells infiltration in the tissue. It was reported that *A. alata* can be found in the small intestine of carnivores and cause inflammation, and cystic lesion with inflammation in arterioles (30). Mild intestinal lesion is caused by *T. cati,* and inflammatory cell infiltration, epithelial destruction and necrosis of tissue may occur (31). The reasons for these changes in small intestine could be pathogenicity of parasites and depression of auto immune system of host (presence of leukocytes) caused by respond of the host body to parasitic infection.

## Conclusion

The present study reported *T. cati, A. alata, and M. lineatus* as the endoparasites of jungle cats studied in the north of Iran, and also showed that these parasite could have histopathologic effects on the small intestine tissue.
